# Electrospun Biomolecule-Based Drug Delivery Systems

**DOI:** 10.3390/biom13071152

**Published:** 2023-07-20

**Authors:** Deng-Guang Yu, Chang Huang

**Affiliations:** School of Materials and Chemistry, University of Shanghai for Science and Technology, 516 Jun-Gong Road, Shanghai 200093, China

Drug delivery, mainly a professional term in pharmaceutics, is a field of interdisciplinary intersection and integration [1,2,3]. Besides pharmaceutics, it is also a popular topic in tissue engineering, medical engineering, food engineering, hygiene, agriculture, and forestry [4]. Small drugs are administered directly in a pure state due to a number of reasons such as possible toxicity, effectiveness, and patients’ compliance. Drug delivery often involves the reasonable selection of drug carriers and additives, which are expected to safely escort the drug molecules to the lesion site (the right section of Figure 1). Thus, two issues are naturally the key elements in developing novel drug delivery systems (DDSs). One is the properties of a drug carrier and its advantages as a drug-loaded matrix. The other is the material conversion techniques by which the drug, the carrier, and the additive are processed into a suitable DDS.

Polymers and lipids are the most important drug carriers both in pharmaceutical industries and laboratories [5,6,7]. Other carriers include inorganic materials (such as silicon, carbon nano tubes, and graphene), surfactants, and organic–inorganic composites (such as MXene and metal–organic frames). During the past several decades, the synthetic polymers that have been approved by the FDA for pharmaceutical applications are extremely limited, although petroleum-based materials are booming (such as polyester fiber, polyamide fiber or nylon, and polyacrylonitrile). The mainstream of pharmaceutical excipients is always natural polymers and their derivatives (such as cellulose, polysaccharides, proteins, and nucleic acids) [8,9,10]. Due to being originally derived from biomass, natural polymers always exhibit fine biocompatibility, low toxicity, and biodegradability [11,12]. These natural polymers belong to the category of biomacromolecules, which can be extracted from plants, animals, and microorganisms.

Biomolecules can be categorized according to their molecular weights, i.e., biomacromolecule and small molecules (such as those typically smaller than 500 Da). Furthermore, these biomacromolecules and small biomolecules can be divided into three categories according to their potential applications, i.e., inert, positively active, and negatively active (or toxic) (the left section of Figure 1). In terms of the contribution of biomolecules to drug delivery, half of the history of pharmaceutics is the history of the usage of biomolecules. First of all, there are numerous active pharmaceutical ingredients (APIs) that are small biomolecules (such as the famous curcumin and quercetin for antibacterial applications, paclitaxel for anti-tumor performances, helicid for calming down emotions, and artemisinin as the most effective drug for treating malaria resistance) or biomacromolecules (such as penicillin, insulin, calcitonin, and many other active proteins). They are positively active ingredients that can be achieved from biomass. Certainly, there are also some negatively active ingredients regardless of small molecules or macromolecules. They are toxic and should be avoided from human beings, such as highly toxic Aflatoxin B1 [13,14], snake venom and various other animal and plant toxins. Secondly, inert small biomolecules are frequently explored as drug delivery carriers. One of the famous small molecules frequently utilized for drug delivery are lipids. They are the main component of the cell membrane, and are thus highly biocompatible and nontoxic. Because of their excellent lipophilic characteristics, the trans-membrane properties should be remarkably increased for drug absorbance when it is encapsulated into a lipid product. Meanwhile, lipid coating can also be explored for an improved drug sustained release profile [15].

Thirdly, inert biomacromolecules are popular in developing a wide variety of novel DDSs. Compared with petroleum-based synthetic polymers, inert biomacromolecules have obvious advantages for carrying drugs and for the related drug delivery applications [16]. Some typical inert biomacromolecules are the most brilliant stars in developing novel DDSs, such as cellulose, chitosan, zein, silk fibroin, gelatin, hyaluronic acid, and collagen [17,18,19]. These inert biomacromolecules are not only biocompatible and safe for usage, but also have excellent processability [20]. Drugs and inert biomacromolecules can be transferred into almost all kinds of formats at a wide scale, such as traditional tablets, capsules, microparticles, nanoparticles, nanofibers, and their combinations such as microparticles-on-a-nanofiber and beads-on-a-string products [21,22].

In this nano era, the amount of research on nano products is tremendous (in Web of Science, there are 1,558,808 items when “nano” is utilized as a topic to search at https://www.webofscience.com/wos/alldb/basic-search (accessed on 14 July 2023)). However, commercial nano products are still very limited. The reasons are complex, including but not limited to the following: (1) their complex production processes, regardless of “bottom-up” or “top-down” methods for nanofabrication; (2) their poor robustness and continuity; (3) the few reasonable evaluation methods and standards of the commercial products; (4) the limited alternative raw materials. Particularly notable is the fourth one; facing the serious situation of continuous depletion of fossil resources and increasing environmental pollution, a shift towards environmentally friendly and efficient renewable biomolecules as raw materials is an inevitable trend. For medical applications such as drug delivery, this trend is even more important. It is anticipated that the combination of biomolecules and electrospinning will provide potential useful solutions for these issues, and thus strengthen the drug delivery applications of biomolecule-based nano products.

Electrospinning, as a typical top-down method for creating nano products characterized by Taylor cone (the bottom section of Figure 1), has a series of advantages over many other “top-down” and “bottom-up” nanofabrication processes [23,24,25,26]. Catering to the three directions of nano science and engineering, electrospinning is also moving forward along three directions: (1) smaller to picotechnology; (2) more order such as various nanoarrays; (3) more complex structures such as multi-chamber structures and nano devices [27,28]. Among these, the complex nanostructures are a highlight point, regarded as representing the advancements of nano science and engineering [28]. It has been broadly demonstrated during the past several decades that electrospinning can remarkably strengthen the drug delivery applications of biomolecules through the creation of biomolecule-based medicated nanofibers [20]. Accompanying the developments of electrospinning, these strengthening effects could be further enhanced in the future.

In general, DDSs composed of electrospun biomolecule-based nanofibers can be those: (1) containing pharmaceutical active biomolecules (both small biomolecules and biomacromolecules) as the guest ingredients, which are very common in developing medicated nanofibers; (2) containing inert biomacromolecules as the matrices to load drugs; (3) containing inert small molecules as drug carriers or additives in electrospun nanofibers for a better drug delivery effect. As electrospinning progresses, its advantages in promoting the drug delivery applications of biomolecules will be further developed.

Electrospinning can further enrich biomolecule-based nano DDSs from different aspects. First of all, electrospinning is able to greatly increase the numbers of novel DDSs through various effective approaches. Several examples are included as follows: (1) new electrospun biomolecule-based nanocomposites or nanohybrids to take advantages of the small diameter, large porosity, and huge surface properties of nanofibers, in which new types of biomolecules (whether macromolecules or small molecules, pharmaceutically inert or active) are encapsulated as guest ingredients or as the filament-forming matrices; (2) electrospun complex structure-based multiple-functional nano products with one or more biomolecules as the candidates, which include 2-layer or 3-layer core–shell, 2-section or 3-section Janus, and the multiple-chamber combinations of core–shell and Janus as DDSs [29,30,31,32,33,34]; (3) the organization of electrospun biomolecule-based nanofibers in a layer-by-layer manner or with other intermediate products (such as casting films and fabrics) for a better drug delivery application [35]; and (4) advanced trans-scale functional products such as microparticles-on-a-nanofiber hybrid DDSs [36]. Accompanying the developments of these new DDSs, the related process–property and structure–performance relationships can be elucidated to provide new professional knowledge (Figure 1).

Secondly, the most recent developments of electrospinning have revealed that unspinnable fluids can take part in multiple-fluid electrospinning processes [37,38]. Thus, many biomolecules, whether small molecules or macromolecules, can be processed into nanofibers with other matching components [39,40]. Meanwhile, the combinations of electrospinning with other electrohydrodynamic atomization processes or traditional chemical and physical methods are elevated in the literature, which provides numerous opportunities for biomolecules to be incorporated into drug delivery applications, such as the combination of electrospraying and electrospinning [36].

Thirdly, many electrospun nanofibers are approaching industrial production, and commercial products based on the progresses of electrospinning are seeing production on a large scale. Without doubt, these progresses would be conducive to the commercial DDSs of biomolecules in future. Medicated nanoparticles are the most frequently reported nano products for drug delivery [41,42,43]. Compared with nanoparticle-based products, some nanofiber-based products have obvious advantages in medical transitions for potential clinical applications, particularly some membrane-based medical devices such as wound dressings, oral disintegrating films, and smart skin [44,45].

Fourthly, electrospinning can improve biomolecule-based nano DDSs from a social and economic standpoint. Electrospinning is a single-step and straightforward process for creating nano products, which means that its production cost and time consumption are competitive. Particularly, some studies have noted the energy-saving effects of special electrospinning apparatuses [46]. In addition, as a multiple-field interaction direction, electrospun biomolecule-based DDSs contain many useful teaching materials for science education, engineering education, innovation education, and even safety education in higher education institutes and universities [47,48].

In summary, electrospun biomolecule-based nanofibers, as a kind of combined product holding the advantages of both biomolecules and electrospinning, are expected to strongly expand and strengthen the numbers of novel DDSs in the near future. It can be expected that these DDSs would accelerate and strengthen the applications of biomolecules in a number of fields, particularly in pharmaceutics, medical devices, food packaging engineering, tissue engineering, and agriculture and livestock.

## Figures and Tables

**Figure 1:**
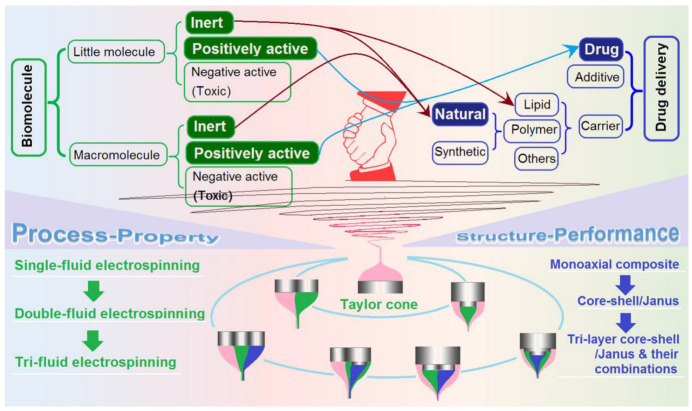
Strengthening the drug delivery applications of biomolecule-based nano products through electrospinning.
